# Epidemiology of Dermatophytoses in Switzerland According to a Survey of Dermatophytes Isolated in Lausanne between 2001 and 2018

**DOI:** 10.3390/jof6020095

**Published:** 2020-06-26

**Authors:** Olympia Bontems, Marina Fratti, Karine Salamin, Emmanuella Guenova, Michel Monod

**Affiliations:** 1 Service de Dermatologie, Laboratoire de Mycologie, Centre Hospitalier Universitaire Vaudois, 1011 Lausanne, Switzerland; Olympia.Bontems@chuv.ch (O.B.); Marina.Fratti@chuv.ch (M.F.); Karine.Salamin@chuv.ch (K.S.); Emmanuella.Guenova@chuv.ch (E.G.); 2 Faculty of Biology and Medicine, University of Lausanne, 1015 Lausanne, Switzerland

**Keywords:** Trichophyton, Microsporum, Epidermophyton, epidemiology, dermatophytosis onychomycosis, tinea pedis

## Abstract

Dermatophytes are the most common pathogenic agents of superficial mycoses in humans and animals. Knowledge of their epidemiology can facilitate the prevention of dermatophytosis and improve prophylactic measures. We sought to determine the incidence of the different dermatophyte species diagnosed in Lausanne (Switzerland) from 2001 to 2018. In total, 10,958 dermatophytes were isolated from patients and 459 from pets. Overall, 99% of tinea unguium and tinea pedis were caused by *Trichophyton rubrum* and *Trichophyton interdigitale* with a prevalence ratio of 3:1. *Trichophyton violaceum* and *Trichophyton soudanense* were mainly found in tinea capitis in patients of African and Mediterranean origin. Interestingly, while *Epidermophyton floccosum* and *Trichophyton verrucosum* were prevalent 50 years ago in an epidemiological analysis carried out in the same laboratory from 1967 to 1970, these two species were rarely detected from 2001 to 2018. *Trichophyton mentagrophytes*, *Trichophyton benhamiae* and *Microsporum canis* were the prevalent zoophilic pathogenic species in children and young adults. Our investigation of animal samples revealed the main reservoirs of these zoophilic species to be cats and dogs for *T. mentagrophytes* and *M. canis*, and Guinea pigs for *T. benhamiae*. This study provides an epidemiological overview of dermatophytoses in Switzerland to improve their surveillance.

## 1. Introduction

Most superficial mycoses are caused by dermatophytes, which infect the stratum corneum of the epidermis, nails and hair. Healthy individuals usually develop a dermatophyte-induced infection at least once during their lifetime, and dermatophytosis is a daily encounter for dermatologists. Accurate dermatophyte identification at the species level is important for both tracking the source of infection and for the initiation of an appropriate treatment [1,2,3]. For example, while the cure rate for tinea capitis caused by the anthropophilic species *Trichophyton violaceum* and *Trichophyton soudanense* is excellent with systemic terbinafine, griseofulvin remains the treatment of choice for other scalp ringworms. Especially, tinea capitis caused by the anthropophilic *Microsporum audouinii* and those caused by zoophilic dermatophytes such as *Microsporum canis* and *Trichophyton mentagrophytes* were revealed to be insensitive to terbinafine [1,2,3].

The prevalence of dermatophyte species continuously evolved from the middle of the 19th century until the present day, and varies depending on the geographic location of the populations [4,5]. The evolution of urban and rural populations, the migration to and from different continents, changes in the human lifestyle, the increasing number of pets and the medical approach to dermatophytosis in different healthcare systems are all factors that account for the observed variable prevalence of dermatophytes amongst different geographical locations. The assessment of the prevalence of pathogenic dermatophyte species and related clinical manifestations is essential and indispensable for good epidemiological surveillance.

Here, we present a comprehensive analysis on all dermatophyte species isolated from human skin and cutaneous appendage lesions referred to the mycology laboratory of the Department of Dermatology at the University Hospital in Lausanne from 2001 to 2018. Additionally, all dermatophyte species isolated from animal lesions and referred to the same department between 2008 to 2018 were included in the analysis. This large data collection, spanning over almost two decades, reveals the current trends in the epidemiology of dermatophyte infections in Switzerland, with a major focus on onychomycosis and common reservoirs of zoophilic fungal species. Comparison of the data with an epidemiological analysis carried out in the same laboratory from 1967 to 1970 showed a change in the prevalence of dermatophyte species in Switzerland over the last 50 years.

## 2. Material and Methods

### 2.1. Processing of Clinical Samples

Skin, nail and hair samples were obtained from patients presenting lesions clinically suspected of fungal infections. All patients consulted a hospital-based physician or a private practitioner in the south-western or Italian part of Switzerland who referred the dermatological samples for diagnostic analysis to our laboratory. Since 2008, animal samples, mainly hair and/or skin scales, have been included in the analysis as well. First, a part of each sample was used for immediate direct mycological examination by fluorescence microscopy [6,7]. In parallel, two culture assays in test tubes were set up. The first tube contained Sabouraud’s agar medium with chloramphenicol (50 μg/mL), and the second tube contained Sabouraud’s agar medium with chloramphenicol plus cycloheximide (400 μg/mL). The inoculated tubes were incubated at 30 °C. Dermatophytes and moulds were identified after 10–14 days of growth by macroscopic and microscopic examination [8,9]. 28S rRNA gene and ITS sequencing were performed as previously described [10,11] when dermatophyte species in cultures could not be identified with certainty based exclusively on their morphological appearance. All data were stored in a MOLIS laboratory information system (CompuGroup Medical [CGM] Lab Belgium NV, Barchon, Belgium).

### 2.2. Nomenclature for Dermatophyte Species Used in This Analysis

The results from our analysis follow the nomenclature adopted in the recent revision of the taxonomy of dermatophytes, and supported by an international expert group of authors [12]. Based on previous studies [10,13], *Trichophyton interdigitale* was used for isolates of tinea pedis and tinea unguium, while *T. mentagrophytes* was used for isolates from another location (mainly tinea capitis and tinea corporis). Mating experiments as well as 28S and ITS sequencing unambiguously allowed the distinction of these two species causing distinct dermatophytoses. The closely related geophilic species *Nannizzia gypsea* (formerly *Microsporum gypseum*), *Nannizzia fulva* and *Nannizzia incurvata*, all three producing numerous spindle-shaped macroconidia [14], were not distinguished. The first name was used for the analysis reports.

## 3. Results

### 3.1. Dermatophytes Isolated from Patients

In total, 77,716 dermatological samples were referred to our laboratory for mycological analysis from 2001 to 2018. The number of isolated dermatophytes was 10,958. Yeasts and moulds were found in 6486 and 8945 culture assays, respectively (Table 1). In onychomycosis, non-dermatophytic fungi may be infectious agents or transient contaminants. Noteworthily, *Fusarium* spp. and *Acremonium* spp. were revealed in 76% of cases when they grew as single species in culture assays [7].

The prevalence of isolated dermatophyte species and their predilection for certain body areas is shown in Table 1. Tinea unguium and tinea pedis were almost exclusively caused by *Trichophyton rubrum* and *T. interdigitale* with a prevalence ratio of about 3:1. Due to the high number of patients referred to our department of dermatology for onychomycosis, *T. rubrum* and *T. interdigitale* were the most frequently isolated dermatophytes (61.2% and 20.1% of the total of dermatophytes, respectively). Other anthropophilic species (*Trichophyton tonsurans*, *T. violaceum*, *T. soudanense*, *M. audouinii*, and *Epidermophyton floccosum*) were isolated with a frequency established between 0.4 and 2.2%. *T. violaceum*, *T. soudanense* and *T. tonsurans* were mainly from tinea capitis. Patients with *T. violaceum* and *T. soudanense* were generally patients originating from Africa and the Mediterranean rim. The anthropophilic species *Trichophyton schoenleinii*, *Trichophyton concentricum* and *Microsporum ferrugineum* were never detected in the patient cohort.

Zoophilic species rarely caused lesions other than tinea corporis and tinea capitis. *T. mentagrophytes*, *M. canis* and *Trichophyton benhamiae* were the most frequently isolated. Their frequency overpassed that of *T. verrucosum*, which was mainly found in the rural population and isolated from tinea capitis, tinea corporis and tinea barbae. *Trichophyton erinacei* and *Nannizzia persicolor* were isolated once and five times, respectively.

Geophilic dermatophytes of the *Nanizia gypsea* group of species (*n* = 55) were uncommon.

### 3.2. Dermatophytes Isolated from Animals

459 dermatophytes were isolated from animal samples collected from 2008 to 2019 (Table 2) *T. mentagrophytes* was the most frequently isolated species from cats and dogs with a prevalence of 62.8% (*n* = 169/269) and 61.0% (*n* = 86/141), respectively. *M. canis* was less often isolated in these hosts with a prevalence of 36.8% (*n* = 99/269) and 23.4% (*n* = 33/141), respectively.

Overall, 35 among 36 dermatophytes isolated from Guinea pigs were *T. benhamiae*. This species was isolated four times from dogs and once from a cat, once from a rabbit and once from a rodent (degu). *T. erinacei* was isolated from a hedgehog and from a skin lesion of its owner, which resulted clinically in a highly inflammatory ringworm on the hand [16].

Species of the genus *Nannizzia* (*N. persicolor* and *N. gypsea*) were almost exclusively isolated from dogs.

Samples from livestock were generally not sent to our laboratory, which explains the low number of isolated *T. verrucosum*.

## 4. Discussion

The present study provides insights into the prevailing dermatophytes in Switzerland. It reveals the dominance of *T. rubrum* as reported in Europe since the nineties [4,5,17,18], the incidence of *T. violaceum* and *T. soudanense* causing tinea capitis in patients of African and Mediterranean origin (mostly immigrants), and the appreciable frequency of *T. mentagrophytes*, *T. benhamiae* and *M. canis* as zoophilic species in the young Swiss autochthonous population.

The prevalence of anthropophilic dermatophytes has changed considerably since the beginning of the 20th century. *T. rubrum*, which was first described in 1910 (as *Epidermophython rubrum*) has developed during the second half of this century to become the dominant species causing dermatophytosis. On the other hand, the anthropophilic species *M. audouinii*, *E. floccosum* and *T. schoenleinii* were the main dermatophytes in the 19th and early 20th centuries, but their frequency has considerably decreased [4,5]. We compared the occurrence of dermatophytes and different ringworms from the present survey with data collected from 1967 to 1970 and from 1990–2000 in the same laboratory [17,19] (Table 3). While *E. floccosum* was still prevalent in the 1960s, representing 17% of dermatophytes isolated, this species now represents less than 0.5% in Lausanne. The incidence of *M. audouinii* was already low in the 1960s (Table 3). Furthermore, a marked difference in the prevalence of the zoophilic species *T. verrucosum* was observed. Whereas 10% of dermatophytes isolated from 1967 to 1970 in Lausanne were *T. verrucosum*, the current analysis documents only 1% of this species among all isolated dermatophytes. The diminution of the incidence of *T. verrucosum* can be interpreted considering the decrease in the rural population. Regarding *T. tonsurans*, its frequency has always been low in Lausanne (about 1%), while this species is prevalent in the USA [4]. To date, we have not registered any Indian patients with tinea corporis or tinea cruris caused by *T. mentagrophytes* type VIII. This particular genotype, which has reached European countries, is responsible for the current epidemic of tinea cruris and tinea corporis in India, where many cases are resistant to terbinafine [20].

*T. benhamiae* was not recorded in the two previous surveys in Lausanne as elsewhere in the 20th century. This species, first described in 1967 by Ajello and Cheng [21] and clearly distinguished from *T. mentagrophytes*, was reported in our laboratory for the first time in 2003 [22]. With the increased number of Guinea pigs and rodents as domestic animals, *T. benhamiae* is now widespread in Europe and Japan [23].

Although human dermatophytoses are predominantly caused by anthropophilic species, zoonotic infections form a significant proportion of cases with *T. mentagrophytes*, *M. canis*. and *T. benhamiae* (Table 2). *T. mentagrophytes* was more prevalent than *M. canis* in contrast to most reports in southern Europe, where the latter is the main zoophilic species and causative agent of tinea capitis and tinea corporis [4,24,25,26]. The investigation of dermatophytes from animal samples showed that cats and dogs were reservoirs for *T. mentagrophytes* and *M. canis*, and Guinea pigs for *T. benhamiae* (Table 3). Cats with *M. canis* are usually domestic indoor cats while hunting cats, mainly European short-haired cats, and dogs are the reservoir of *T. mentagrophytes* [27]. Cats and dogs are most probably colonized or infected with *T. mentagrophytes* through contact with soil. It should be noted here that *T. mentagrophytes* encompasses various genotypes, which were not identified in our routine analyses. While several genotypes of *T. mentagrophytes* correspond to zoophilic dermatophytes identified in cats, dogs and rodents, a recent study in Germany has revealed that isolates of *T. mentagrophytes* causing pubic infections had a particular genotype (type VII), for which no animal source has been found so far. It is therefore possible that the cases of tinea cruris reported in this study are not of animal origin but of human origin [28].

Dogs have also been found to be infected with *N. gypsea* (geophile) and *N. persicolor*, which is considered a zoophilic species because it has been isolated from voles and shrews [12]. However, it is also very possible that the infection of dogs with *N. persicolor* could occur through contact with soil. The origin of *T. erinacei* in a patient could be traced to contact with a hedgehog [16], as was the case for several cases of this emerging dermatophyte species in Spain and Germany [29,30,31,32].

Knowledge of the epidemiology of dermatophytes can facilitate the prevention of dermatophytosis and intervention with prophylactic measures. Zoophilic dermatophytes apparently lose their pathogenicity and are less contagious after infecting humans [33], and most infections are acquired directly from animals. Therefore, the best preventive measure to avoid infection with a zoophilic species is to avoid direct contact with pets. It is important to accurately identify the fungus involved in inflammatory dematophytosis and to carefully examine pets as a possible source of infection.

## Figures and Tables

**Table 1:** Prevalence of different fungi in dermatological samples analyzed in this study.

Fungi Identified by Cultures	Scalp	Face	Perineum	Hand	Body	Foot	Nail	Total	Percent ^2^
	(Tinea Capitis) ^1^	(Tinea Faciae) ^1^	(Tinea Cruris) ^1^	(Tinea Manum) ^1^	(Tinea Corporis) ^1^	(Tinea Pedis) ^1^	(Tinea Unguium) ^1^		
Dermatophytes									
Anthropophilic species									
*T. rubrum*	13	38	338	100	259	1546	4413	6707	61.21
*T. interdigitale*	0	0	0	0	0	862	1345	2207	20.14
*T. violaceum*	213	12	1	0	12	3	3	244	2.22
*T. soudanense*	134	3	3	3	28	5	21	197	1.8
*T. tonsurans*	71	17	1	2	29	1	1	122	1.11
*E. floccosum*	1	1	6	1	10	11	10	40	0.37
*M. audouinii*	144	6	0	1	15	0	3	169	1.54
Zoophilic species									
*T. mentagrophytes*	86	119	67	39	279	0	0	590	5.38
*T. benhamiae*	19	39	0	11	68	2	0	139	1.27
*T. verrucosum*	27	24	1	6	49	4	2	113	1.03
*T. erinacei*	0	0	0	1	0	0	0	0	0.01
*M. canis*	116	23	7	3	217	2	1	369	3.37
*N. persicolor*	0	1	0	0	3	0	1	5	0.05
Geophilic species									
*N. gypsea*	6	0	3	3	37	3	3	55	0.5
Total dermatophyte cutures	830	283	427	169	1006	2439	5803	10,958	*14.1*
Other fungi without dermatophytes									
Yeasts	54	693	1421	212	205	603	3298	6486	*8.35*
*Fusarium* spp./*Acremonium* spp.	2	2	2	2	3	70	1078	1159	*1.49*
Other moulds	219	112	73	255	359	931	6996	8945	*11.51*
Total positive fungal cultures	1105	1090	1923	638	1573	4043	17,175	27,547	*35.45*
Negative fungal cultures	3149	1734	4424	1997	5908	6801	26,155	50,168	*64.55*
Total number of samples	4254	2822	4072	2634	7472	10,844	49,133	77,716	*100*

^1^ Dermatophyte infections. ^2^ Percentages in relation to the number of dermatophytes (*n* = 10,958) are in straight line characters. Percentages in relation to the total number of samples analyzed (*n* = 77,716) are in italics.

**Table 2:** Dermatophytes isolated in Lausanne from animal samples collected by veterinarians between 2008 and 2019.

	Cats	Dogs	Guinea Pigs	Rabbits	Horses	Miscellaneous	Total
*T. mentagrophytes*	169	86	1	2	1 ^1^		259
*M. canis*	99	33				1 (Cheetah)	133
*T. benhamiae*	1	4	35	1		1 (Degu)	42
*N. persicolor*		6					6
*N. gypsea*		12			2		14
*T. verrucosum*						1 (Cattle); 1 (Swines)	2
*T. equinum*					2		2
*T. erinacei*						1 (Hedgehog)	1
Total	269	141	36	3	5	5	459

^1^ Published as a case report [15].

**Table 3:** Prevalence of dermatophyte species (%) from three different surveys during the last 50 years in Lausanne.

	1967–1970 Gregoriu and Brot, 1972 [19] *n* = 584	1990–2000 Monod et al. 2001 [17] *n* = 4193	2001–2018 This Study *n* = 10,958
Anthropophilic species			
*T. rubrum*	35.1	62	60.07
*T. interdigitale*	30.9 ^1^	15.6	20.14
*T. tonsurans*	0.9	0.1	1.12
*T. violaceum*	0.2	1.7	2.29
*T. soudanense*	0.4	1.6	1.85
*T. schoenleini*	0.5		
*E. floccosum*	17	1	0.36
*M. audouinii*	0.4	2.3	1.68
*M. ferrugineum*	0.4		
Zoophilic species			
*T. verrucosum*	10.2	1.3	1.03
*T. mentagrophytes*	30.9 ^1^	9.9	5.38
*T. benhamiae*			1.27
*T. quinckeanum*	0.2		
*T. equinum*			
*T. erinacei*			
*M. canis*	2.4	5	3.51
Geophilic species			
*N. gypsea*	2.5	0.2	0.52
*N. persicolor*	0.2	0	0.05

^1^ T. interdigitale and T. mentagrophytes were not differentiated.
